# A case report of a cystic fibrosis patient with repeated isolation of *Trichosporon mycotoxinivorans* identified by a novel short-extraction method

**DOI:** 10.1186/s12879-016-1910-7

**Published:** 2016-10-25

**Authors:** Daniel Goldenberger, Vladimira Hinić, Spasenija Savic Prince, Michael Tamm, Anna-Maria Balestra, Doris Hohler, Reno Frei

**Affiliations:** 1 Division of Clinical Microbiology, University Hospital Basel, Petersgraben 4, CH-4031 Basel, Switzerland; 2 Division of Pathology, University Hospital Basel, Schönbeinstrasse 40, CH-4031 Basel, Switzerland; 3 Division of Pneumology, University Hospital Basel, Petersgraben 4, CH-4031 Basel, Switzerland; 4 Present address: Division of Pneumology, St. Claraspital Basel, Basel, Switzerland

**Keywords:** *Trichosporon mycotoxinivorans*, Cystic fibrosis (CF), Internal transcribed spacer (ITS), Emerging pathogen, MALDI-TOF MS

## Abstract

**Background:**

*Trichosporon mycotoxinivorans* is a recently described yeast-like fungal organism and its association as a pathogen for patients with cystic fibrosis (CF) was reported previously. We show the clinical course of a CF patient over 9 years as well as the applications of modern molecular and proteomic identification techniques of this rare fungus.

**Case presentation:**

We present the case of a 32-year-old male CF patient with sputum cultures continuously positive with the anamorphic yeast *T. mycotoxinivorans* during 9 years. Furthermore, susceptibility testing of *T. mycotoxinivorans* to different antifungals were performed. In addition, a rapid identification method of this novel fungal pathogen with matrix-assisted laser desorption ionization-time of flight mass spectrometry (MALDI-TOF MS) was applied using a simple extraction protocol.

**Conclusions:**

Our case presentation confirms *T. mycotoxinivorans* as a potential emerging pathogen in patients with CF. However, our CF patient showed mild symptoms over a very long time period of 9 years. A short MALDI-TOF MS procedure allows reliable and rapid identification of *T. mycotoxinivorans* and therefore should facilitate further study on the clinical relevance and epidemiology of this unusual fungal organism.

## Background

The genus *Trichosporon* comprises 35 species of this basidiomycetous yeast from which 6 are known to be medically-relevant mainly in the immunocompromised patient [1, 2]. They are ubiquitous yeasts, found in soil and water and known as colonizers of skin and gastrointestinal tract of humans [1]. *Trichosporon mycotoxinivorans* was described for the first time in 2004, when it was isolated from the hindgut of the lower termite *Mastotermes darwiniensis* (*Mastotermitidae*) [2]. The name of this species refers to the ability of *T. mycotoxinivorans* to degrade certain mycotoxins, an important characteristic which could be used for deactivation of the respective mycotoxins in animal feeds [2]. Its association with cystic fibrosis (CF) patients was reported for the first time in the USA in 2009 [3]. A 20-year-old male with CF died with histologically proven *T. mycotoxinivorans* pneumonia. Another case of a fatal disseminated coinfection with *T. mycotoxinivorans*, *Aspergillus fumigatus* and *Scedosporium apiospermum* in a CF patient, recipient of lung and liver transplant, was published recently [4]. Kröner et al. demonstrated the potential association of *Trichosporon* spp. with severe exacerbations in CF patients [5]. On the other hand, the results of the most recent study by Shah et al. showed that the isolation of *T. mycotoxinivorans* from CF patients is not necessarily associated with significant changes in clinical status [6]. Here we report on a CF patient with repeated isolation of *T. mycotoxinivorans* in respiratory specimens during the past 9 years.

## Case presentation

A 32-year-old male was regularly followed in our clinic because of CF. His lung function had been stable and is slowly decreasing since the last 7 years with a moderate irreversible airflow obstruction with an FEV1 of 60 % (predicted). He was known to be suffering from a chronic colonization with *Staphylococcus aureus*, *Stenotrophomonas maltophilia*, and *Pseudomonas aeruginosa* for which he has been treated repeatedly with oral antibiotics (trimethoprim/sulfamethoxazole, doxycycline and amoxicillin/clavulanic acid) as well as rarel intravenous regimens with piperacillin/tazobactam and tobramycin. He was put under permanent treatment with azithromycin and intermittent adjunct of amikacin for inhalation. Further he had a history of allergic bronchopulmonary aspergillosis leading to a subacute clinical deterioration. He was successfully treated with oral and inhaled steroids.

**Fig. 1:**
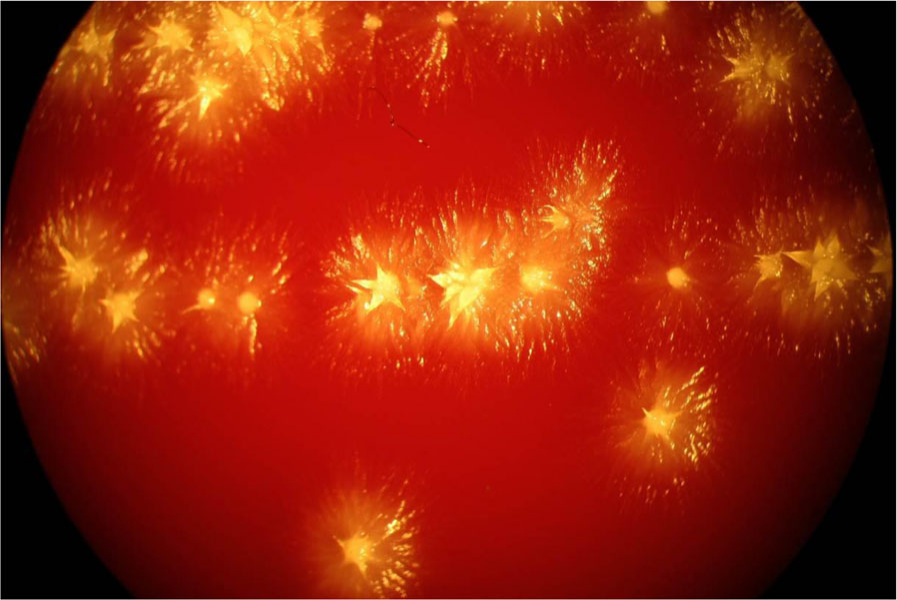
Colonies of *T. mycotoxinivorans* on sheep blood agar, 40-fold magnification

**Fig. 2:**
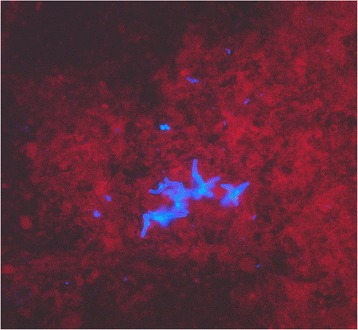
Fluorescence microscopy of bronchoalveolar lavage sample; Fungiqual A, 400-fold magnification

**Fig. 3:**
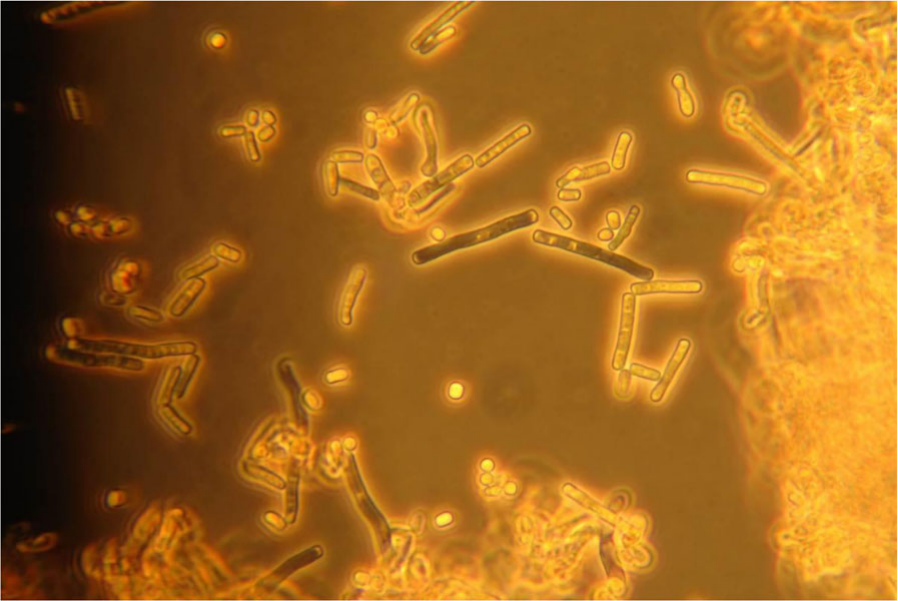
Microscopy of *T. mycotoxinivorans* showing arthroconidia, 400-fold magnification

In 2007, a basidiomycetous yeast was isolated from sputum of the patient. The colonies grew after 2 days of incubation on Sabouraud agar (Becton Dickinson Diagnostic Systems, Allschwil, Switzerland), showing typical “furry” appearance (Fig.1) and histopathology of the bronchoalveolar lavage specimen showed not-specified fungal structures (Fig. 2). The organism was identified as *Trichosporon* species based on phenotypic features such as the typical morphology of colonies with aerial whitish mycelium (Fig. 1) and characteristic arthroconidia (Fig. 3) as well as the use of the API^®^ ID32C identification system (bioMérieux, Geneva, Switzerland). Identification of the *Trichosporon* isolate to the species level was performed in 2011, based on PCR amplification and sequencing of the internal transcribed spacers (ITS), as well as the D1/D2 region of the 26S rRNA gene [7]. The investigated sequence showed 100 % identity to reference sequences of T. *mycotoxinivorans*. Our 1167-bp-long sequence has been deposited in GenBank under accession no. JQ266092. In 2012, matrix-assisted laser desorption ionization-time of flight mass spectrometry (MALDI-TOF MS) was performed for the first time from colony material using the full ethanol-formic acid extraction protocol as recommended by the manufacturer. The analysis on the MALDI BioTyper system with the BioTyper 3.0 SR software (Bruker Daltonics GmbH, Bremen, Germany) resulted in highly accurate species identification with scores higher than 2.200. The manufacturer recommends ≥2 log score for species identification; 1.7 to 1.99 for genus identification; and <1.7 as unreliable [8]. Subsequent isolates were tested with a simple “short extraction” protocol, consisting of addition of 1 μl of 70 % formic acid to the smears on the plate before application of the matrix solution [9]. The “short extraction” yielded scores ranging from 1.866 to 2.136 (average 1.989, median 1.984). The organism was isolated repeatedly from respiratory specimens during the period from 2007 to 2016. An overview of the microbiological results is shown in Table 1. The susceptibility testing of the strain was performed in 2012 and 2013 using a commercial microtitre system (YeastOne, Trek Diagnostic Systems, Thermo Fisher Scientific Schweiz AG, Reinach, Switzerland). The results of the susceptibility testing are shown in Table 2. The strain exhibited high level resistance to the echinocandins (anidulafungin, micafungin, caspofungin) and variable susceptibility to amphotericin B and the triazoles (fluconazole, itraconazole, voriconazole and posaconazole). Due to further clinical deterioration, an antifungal therapy with voriconazole was initiated (200 mg twice a day). Clinical amelioration was observed within 6 weeks. However, a clinical relapse occurred after 9 months later and *T. mycotoxinivorans* was again identified. After detection of a large quantity of two morphotypes of *Trichosporon mycotoxinivorans* the therapy was changed to itraconazole with a very good clinical response and lung functional response. Over the years the clinical course was characterized by recurrent worsening of the respiratory situation expressed by a mostly mild symptomatology with a marked reduction of the detectable *T. mycotoxinivorans* quantity and satisfying stable clinical results after the treatment with triazoles. It is possible that by using voriconazole, *A. fumigatus* has been suppressed together with *T. mycotoxinivorans*. There might have been also a reduction of the antigenic stimulus for bronchial inflammation as it has been seen in therapies with itraconazole. Because of the stability of the clinical course after the treatment with voriconazole, a long term suppressive therapy with antifungal agents was not applied.

**Table 1:** Chronology of microbiological findings in the sputum samples of the patient

Year	Organisms isolated, amounts^a^
2007	*Staphylococcus aureus*, +++
	*Stenotrophomonas maltophilia*, +++
	*Aspergillus fumigatus*, ++
	*Trichosporon* sp., ++
2008	*Staphylococcus aureus*, ++
	*Stenotrophomonas maltophilia*, +
	*Aspergillus fumigatus*, +
	Oral flora, ++
	*Trichosporon sp.*, +
2009	*Staphylococcus aureus*, +++
	*Stenotrophomonas maltophilia*, ++
	Oral flora, +++
	*Trichosporon* sp., ++
2010	*Staphylococcus aureus*, ++
	*Stenotrophomonas maltophilia*, +++
	Oral flora, ++
	*Trichosporon* sp., ++
2011	*Stenotrophomonas maltophilia*, ++
	*Aspergillus fumigatus*, ++
	Oral flora, ++
	*Trichosporon mycotoxinivorans*, +++
2012	*Staphylococcus aureus*, ++
	*Stenotrophomonas maltophilia*, ++
	*Pseudomonas aeruginosa*, +
	*Trichosporon mycotoxinivorans*, +
2013	*Staphylococcus aureus*, +++
	*Stenotrophomonas maltophilia*, +
	Mould, not identified, +
	Oral flora, +
	*Trichosporon mycotoxinivorans*, +
2014	*Staphylococcus aureus*, +++
	*Haemophilus haemolyticus*, +++
	Oral flora, +++
	*Trichosporon mycotoxinivorans*, +
2015	*Staphylococcus aureus*, ++
	Oral flora, ++
	*Trichosporon mycotoxinivorans*, ++
2016	*Staphylococcus aureus*, ++
	Oral flora, ++
	*Trichosporon mycotoxinivorans*, ++

^a^Amounts: +, few; ++, moderate; +++, numerous

**Table 2:** Antifungal susceptibility testing of *T. mycotoxinivorans* isolated in 2012 (Morphotype 1 and Morphotype 2) and 2013

	s503348/2012	s503348/2012	s503026/2013
	Morphotype 1	Morphotype 2	
Antifungal agent	MIC^a^	MIC^a^	MIC^a^
Amphotericin B	0.5	2.0	0.25
Anidulafungin	>8.0	>8.0	>8.0
Micafungin	>8.0	>8.0	>8.0
Caspofungin	>8.0	>8.0	>8.0
Flucytosine	16	32.0	64
Posaconazole	0.06	0.25	0.12
Voriconazole	0.03	0.12	0.03
Itraconazole	0.015	0.25	0.03
Fluconazole	2.0	8.0	4.0

^a^ *MIC* minimal inhibitory concentration expressed in mg/l

The systematic search for *Trichosporon* colonization in our microbiological data sheets of 64 CF patients, 10 patients after lung transplantation, and 32 patients with bronchiectasis did not reveal any other patients with *Trichosporon* spp.

In addition to arthroconidia, *T. mycotoxinivorans* may sometimes microscopically display large fusiform cells with granular contents called fusiform giant cells [10]. This feature, however, should not be used for species identification, since fusiform giant cells may also appear in other *Trichosporon* spp. like *T. loubieri* and *T. vanderwaltii* [10, 11]. Furthermore, in some *T. mycotoxinivorans* isolates like the one described in this case report or communicated by Hickey et al. [3], the fusiform giant cells may not be present. This feature is probably culture medium or culture age dependent. To date, the only reliable identification of this yeast to species level could be achieved by sequencing, available only in specialized clinical laboratories. The MALDI-TOF MS identification is becoming the standard for isolate identification in many laboratories. In contrast to Hirschi et al. [4], we were able to reliably identify the *Trichosporon* yeast to the species level using the MALDI-TOF MS identification. Our findings are in congruence with successful identification of this pathogen by Hickey and colleagues [3]. However, in our study, we describe for the first time the identification of this yeast by using the “short extraction” on-plate protocol for the routine diagnostics. As the scores obtained were somewhat lower than those with the full extraction protocol, we propose using lower species cutoff values for identification of *T. mycotoxinivorans*. Our proposal is consistent with studies of Vlek and Van Herendael [9, 12], which consider a species cutoff value of as low as 1.7 as appropriate for routine yeast identification in clinical laboratories. MALDI-TOF MS, as a rapid and reliable identification tool may facilitate further study on the reservoir and the epidemiologic link of *T. mycotoxinivorans* to CF patients. Concerning its antifungal susceptibility, the genus *Trichosporon* presents increased in vitro resistance to common antifungal agents such as amphotericin B and echinocandins [1, 2], but also strains resistant to multiple triazoles have been identified [3]. Due to variable susceptibility patterns to triazoles, which are considered as therapy of choice [3], every strain isolated should be tested for antifungal susceptibility. Attempts to treat our patient with triazoles did not lead to an eradication of *T. mycotoxinivorans*, but to an improvement of the clinical symptoms.

## Conclusions

In summary, our report on a CF patient colonized with *T. mycotoxinivorans* for a long period of 9 years suggests that this yeastlike organism may be associated with clinical deterioration, and the patient’s condition improved under therapy with voriconazole and itraconazole. *T. mycotoxinivorans* could be successfully identified with a simple on-plate MALDI-TOF MS short extraction protocol that may facilitate further study on the reservoir and the epidemiologic link of *T. mycotoxinivorans* to CF patients and immunocompromised persons.

## Abbreviations

CF: Cystic fibrosis
FEV1: Forced expiratory volume in 1 second
MALDI-TOF MS: Matrix-assisted laser desorption ionization-time of flight mass spectrometry
